# Navigating Uncertainty: A Qualitative Study on the Hospitalization Experience of COVID-19 Patients

**DOI:** 10.7759/cureus.72281

**Published:** 2024-10-24

**Authors:** Eleni Tsimitrea, Evdokia Misouridou, Aikaterini Toska, Maria Saridi, Stylianos Boutlas, Ioanna V Papathanasiou, Konstantinos I Gourgoulianis, Evangelos C Fradelos

**Affiliations:** 1 Department of Nursing, University of Thessaly, Larissa, GRC; 2 Department of Nursing, University of West Attica, Athens, GRC; 3 Department of Respiratory Medicine, Faculty of Medicine, University of Thessaly, Larissa, GRC; 4 Department of Respiratory Medicine, Faculty of Medicine, University of Thessaly, University General Hospital of Larissa, Larissa, GRC

**Keywords:** covid-19, fear, hospitalization, isolation, support, uncertainty

## Abstract

This study examines the hospitalization experience of COVID-19 patients in public hospitals in the Thessaly Region of the Greek territory. The ultimate goal is to explore and gain a deeper understanding of the subjective experience of hospitalization. A qualitative study design was employed in this study. Data were collected through semi-structured interviews with 12 patients. Four categories emerged when analyzing the data: (a) personal experience, (b) the unknown nature of the disease, (c) causal attributions and meaning-making, and (d) mental resilience. The findings underscore the necessity of providing ongoing and comprehensive care for COVID-19 patients that addresses their biological, psycho-emotional, and social needs from the time of diagnosis to full recovery.

## Introduction

The COVID-19 pandemic, as a complex event with long-term and multilevel impacts, is a part of several health crises that have as a common denominator, in addition to direct and/or chronic public health impacts, significant disruptions in the mental well-being of people worldwide [1]. The root cause of the phenomenon is the need to contain the rapid spread of the virus, which led to the implementation of a wide range of preventive health and severe containment measures, including isolation and social distancing as the main strategies [2]. Although the abovementioned measures, especially home isolation in combination with the use of personal protective means [3], proved to be highly effective in stopping the transmission of the virus and, by extension, in fighting the pandemic, they had the inevitable consequence of causing social isolation and loneliness of a large part of society, placing a significant psychological burden on the population [4]. The continuous coverage of the pandemic by the mass media, the over-information, and the excessive focus on the detailed cases per region, along with the pressure for vaccination through information campaigns and the presentation of negative consequences of non-vaccination coverage [5], contributed to the strengthening and worsening of the psychological problems of the population. Similar to previous global crises, the combined effect of restrictive measures and over-information concerning the pandemic, while necessary, largely shaped public perceptions of the disease and dramatically accelerated the rate at which people transformed their social interactions and practices, adapting to an ever-evolving world [6].

Established standard practices and habits that were followed daily were called into question. Instead, humanity was forced to adapt to a new lifestyle, characterized by adherence to health protocols and the frequent and correct application of personal hygiene measures. Examples include the thorough washing of hands with soap and water and/or the application of an alcohol-based antiseptic, the use of a suitable (cloth or surgical) protective face mask, the cleaning and disinfection of premises and surfaces at regular intervals, the compulsory laboratory test for the detection of COVID-19 even with a sneeze, and the avoidance of gatherings. As a result, these new conditions in everyday life imposed by the pandemic radically transformed the way humanity perceived hygiene, sociality, and even work [7]. Everyday interpersonal interactions were avoided, the possibility of religious practices was reduced, and the transition to a digital lifestyle (teleworking and tele-education) was imposed. At the same time, major rearrangements occurred in the labor market, with the loss of many jobs and the need for new knowledge and skills increasing the concern for economic security and survival [7,8].

These major shifts caused by the COVID-19 pandemic prompted Gruber and his colleagues [9] to describe the pandemic as one of the greatest challenges for humanity with an impact similar to that of the Spanish flu in the previous century (1918-1920) [10]. States, as then, implemented strict containment measures to contain the dramatic increase in global morbidity and to protect their health systems, partly despite the potential impact on the mental health of a significant part of the population [11]. A study highlighted the significant impact of the COVID-19 pandemic on people's mental health [12], concluding that COVID-19 infection has been the most traumatic and stressful event experienced by modern humans in recent decades. These findings are supported by recent scientific data suggesting that the social distancing measures imposed during the pandemic are directly correlated with the onset of personality, mood, and anxiety disorders [2]. Individuals during this unprecedented health crisis were called upon to deal with a combination of unprecedented challenges and situations that profoundly affected every aspect of human activity. Physical and mental health, social relationships, and the economy were deeply affected [2,13].

It can be effortlessly concluded that the COVID-19 pandemic brought to light vulnerable aspects of societies worldwide, as the threat of COVID-19 and the accompanying containment measures have resulted in a widespread outbreak of population-based problems [12], which were rooted in the intensity of feelings of anxiety, fear of contracting and transmitting the virus, fear of death, shame, mental distress, and the inability to protect and care for themselves and their loved ones [13,14]. Individuals who became severely ill with COVID-19 and were hospitalized in nursing homes experienced particularly traumatic experiences (e.g., pain, difficulty in breathing, and fear of death) and faced the first-hand effects of the disease due to fear of their health outcomes. Additionally, being away from loved ones during hospitalization enhanced the feelings of loneliness and isolation [15].

The experience of hospitalization for patients with coronavirus is characterized by feelings of loneliness, fear, and uncertainty. Patients were isolated from their families due to the strict protocols, which exacerbated their stress [16]. Difficulty breathing and the other serious complications of the disease intensify the feeling of helplessness, while the inability to communicate with staff due to protective measures creates a sense of isolation [17]. According to studies, patients were cut off from support and human contact, while uncertainty about the outcome of the illness causes intense psychological stress. These experiences highlight the need for better communication and emotional support during hospitalization [18].

Although the experience of hospitalization has been studied in international literature, this aspect has not been fully investigated in Greece. By studying the hospitalization experience of this category of patients, the present study aims to analyze the psycho-emotional and social impact suffered by COVID-19 patients during their hospitalization in Greek hospitals.

## Materials and methods

Methodology

Study Design

This qualitative study aimed to examine and highlight the hospitalization experiences and main challenges faced by patients with COVID-19 in public hospitals in Thessaly, with emphasis on the University General Hospital of Larissa and the General Hospitals of Larissa, Volos, Karditsa, and Trikala during the period from January 2023 to January 2024. Semi-structured interviews were used to investigate the attitudes, perceptions, and behaviors of the participants.

Sampling and Participants

A sample of 12 COVID-19 patients who were hospitalized in COVID-19 clinics of public hospitals in Thessaly was purposely selected for this qualitative study. Patients who had been hospitalized due to COVID-19 were included. Patients with a known history of mental or psychological disorders and patients with communication issues such as speech or hearing problems were excluded.

The sample consisted of adults aged 35-78 years (mean age: 55 years), with a slight predominance of women (7 out of 12). The length of hospital stay ranged from 2 to 32 days (mean: 10.6 days). Regarding the severity of the disease, one patient required intubation, while nine required oxygenation. In addition, most participants (8 out of 12) were treated in multiclinic wards, while the rest were treated in single rooms. Detailed patient characteristics are presented in Table 1.

**Table 1:** Participants’ demographic data

Participant code	Gender	Age (years)	Marital status	Educational status	Occupation
P1	F	52	Widow - 2 children	University degree	Retired
P2	F	77	Widow - 1 child	Secondary school	Retired
P3	F	53	Married	High school	Public sector
P4	F	67	Widow - 2 children	Secondary school	Household
P5	F	37	Single	University degree	Private sector
P6	F	66	Widow - 2 children	Secondary school	Household
P7	M	48	Married - 1 child	MSc	Freelancer
P8	M	62	Single	High school	Public sector
P9	M	50	Married - 2 children	High school	Freelancer
P10	M	49	Single	University degree	Public sector
P11	M	52	Married - 2 children	MSc	Military officer
P12	F	53	Married - 1 child	University degree	Private sector

Data Collection Procedure

The present study was conducted in full compliance with international and national regulations governing the ethical conduct of research, and approval was obtained from the Scientific Advisor of the University General Hospital of Larissa.

Before their participation, participants were thoroughly and clearly informed about the study's objectives, the data collection process, and their rights, including the right to refuse participation, the preservation of their anonymity, and the protection of their personal data. Particular attention was paid to informing older participants, considering their intellectual needs, and then written informed consent was obtained from them for their participation in the research.

Data collection was carried out through semi-structured interviews lasting approximately 40 minutes, which were conducted face-to-face. A combination of open-ended and closed-ended questions was used to gain an optimal understanding of the participants' experiences. This was followed by a series of open-ended questions that explored in-depth thoughts, feelings, and specific aspects of their experience before, during, and after their illness (Table 2).

**Table 2:** Interview guide

Interview guide
Could you share your thoughts on how you believe you contracted the coronavirus?
How did you feel when you received a positive COVID-19 test result? What thoughts crossed your mind at that moment?
Were you frightened by the diagnosis?
Before the pandemic, did you have any concerns about being hospitalized? If so, what were they?
When you were admitted to the hospital with COVID-19, did those fears you had before the pandemic intensify?
How would you describe your overall experience of being hospitalized with COVID-19?
How did you spend your time during your stay in the hospital?
Can you describe your emotions during the most challenging part of your illness?
How did you manage to cope with the physical and emotional difficulties you were facing?
Do you think the way the media covered the pandemic influenced your psychological state or how you viewed the disease and its risks?
Were there moments when you felt your life was in danger because of the illness?
What message would you like to convey to others (whether they are patients or loved ones) who are going through a similar experience?

Analysis

To examine the experience of patients with COVID-19 during their hospitalization in public hospitals of Thessaly, an inductive content analysis was applied to analyze qualitative data [19]. The procedure included the following stages [20]: (1) initial familiarization with the data through repeated data reading and open-source coding, (2) organization of codes into categories and subcategories, (3) review, (4) extraction of central topics and creation of a thematic data map, and (5) composition/reporting of data - writing up the findings.

Ethics approval of research

This study has been approved by the internal ethics and ethics committee of the nursing department of the University of Thessaly in Greece (reference number: 978/10.07.2023). The study was conducted by the ethical principles outlined in the Declaration of Helsinki. All participants were informed about the purpose, procedures, and potential risks of the study, and they provided written informed consent. The confidentiality of all patient information was ensured. To protect the anonymity of participants, anonymous identifiers were used, and data were stored on a secure server with encryption. Only authorized personnel had access to the data, which would be retained for two years and then destroyed.

## Results

The results of the data analysis highlight the high degree of diversity in the experiences, attitudes, and perceptions of patients with COVID-19 during their hospitalization in COVID-19 units of public hospitals. Deeper analysis of the data led to a taxonomic system of participants' perceptions and emotions during their hospitalization in COVID-19 units that includes four main categories and 11 subcategories, providing a detailed framework for understanding the psycho-emotional and social impact of the disease (Table 3).

**Table 3:** Categories and subcategories

Categories	Subcategories
Personal experience	Communication with loved ones and emotional connection
	Assistance from health professionals and stigmatizing distinctions
	Impact of social distancing
Unknown nature of the disease	Fears and concerns for children
	Confusion and lack of information
	Anxiety about the future
Causal attributions and meaning-making	Internal attributions
	External attributions
Toward mental resilience	Spirituality
	Personal resources for mental resilience
	Help from health professionals

Category 1: personal experience

This category is divided into three subcategories: (a) communication with loved ones and emotional connection, (b) assistance from health professionals and stigmatizing distinctions, and (c) the impact of social distancing on mental health.

The inability of patients to have face-to-face contact with their relatives due to the strict health protocols applied during their hospitalization forced the participants to rely primarily on new technologies and electronic means (e.g., voice calls, video calls, and messaging via personal mobile phones) to maintain contact with them. However, digital communication, although an important gateway, could not provide high levels of emotional connection or replace the emotional connection offered by physical presence. It is important to note that public hospitals did not provide patients or their relatives with special communication devices, and the COVID-19 wards were also not equipped with a TV and/or radio, which exacerbated the sense of isolation and loneliness of the patients.

“There was none of that in the ward, no TV or radio. There was just a room with four beds. From then on... Basically, I used my mobile phone to check the media and to contact my relatives. They would bring me a newspaper so that I could read during the hours I was alone” (P11).

The attitude of health professionals and the quality of care provided were important aspects of the patient experience. A small number of participants reported that their contacts with health professionals were limited in frequency and duration and that the latter's response to any appeals was not always prompt and in line with their personal needs. Of course, participants' reports varied depending on the individual case, as they were influenced by factors such as the patient's personality, their health status, and their general interaction with health care providers.

“The medical-nursing staff were not willing to help. They came to the ward once or twice a day” (P10).

“They would come into the ward at certain times and sometimes you couldn't be taken care of. They were not always available” (P8).

“They did not always respond to our calls. The staff were generally impersonal, they used to pass by at certain times and provided us with what they thought they should provide” (P3).

A large number of participants primarily attributed the reduced response from health professionals to two main reasons: (a) the extremely demanding working conditions that prevailed (for example, the severe shortage of staff and logistical equipment and/or the heavy workload) and (b) the need for strict adherence to health protocols.

“There was one nurse available” (P12).

“*…the visits by the cleaning crew were minimal. I understand and respect it because of their fear and ignorance. They were getting in contact with us for as little as possible ... the staff was minimal*” *(P10).*

A common ground between vaccinated and non-participants was the fact that the latter were approached differently by healthcare providers. This consisted of providing reduced assistance, guidance, and counseling on one hand while offering minimal psycho-emotional and social support and information on the other.

“Staff behavior was better towards the vaccinated than the unvaccinated” (P8).

“… it wasn't perfect. I noticed better behavior towards the vaccinated than the unvaccinated” (P9).

In this respect, the attitude of health professionals toward vaccinated participants was significantly different. Based on their reports, the behavior of medical and nursing staff was highly supportive (such as increased frequency of visits, provision of personalized care, information, and support).

“I had no complaints from the staff. I was very happy and felt safe. In fact, the doctors - pulmonologists - were passing by to see me in the afternoons” (P4).

In conclusion, the participants noted a delay in the delivery of personal products and essentials (mobile phones, clothes, medicines, books, etc.) that they had requested from their relatives to facilitate their stay at the COVID-19 clinic during their hospitalization.

“Once it happened to ask them for a call. It was done, but much later - after three days, two or three days if I remember correctly” (P3).

“My family sent me some things, but I didn't receive them immediately. I received them the day after” (P10).

The duration of experienced social isolation is potentially associated with participants' impaired mental health. The restriction of freedom of movement and the absence of face-to-face personal contact with their loved ones contributed to the worsening of pre-existing symptoms of the disease, the intensification of psychological distress, and the aggravation of the overall hospitalization experience.

“The isolation was scaring me. My freedom of movement was restricted. I wanted to get out of the ward, and I couldn't... the staff was coming in at certain times wearing their special uniforms. It was scary because I couldn't see who they were” (P8).

“I was afraid of the feeling of being trapped in a certain space” (P7).

Any reported psychological consequences of social isolation (such as loneliness or feelings of reduced social support) were further exacerbated possibly due to zero to minimal social interaction with their relatives and healthcare staff. In contrast, loneliness and sad feelings of detachment from other people, usually accompanied by mental fatigue and bitterness, were reduced in those cases where communication with significant others depended on access to personal technology (e.g., mobile phones).

“There was nothing in the ward, no TV, no radio. There was just a room with four beds. From then on ... I used my mobile phone to read the media and to contact my relatives. They would bring me some newspapers so that I could read the hours I was alone ... I was frightened by the isolation and loneliness” (P11).

Category 2: unknown nature of the disease

This category includes three subcategories: (a) concern and fear for the welfare of children, (b) confusion and lack of information, and (c) fear and anxiety about future situations and events.

Many participants described their hospitalization period to the researcher as a significantly stressful and difficult experience. The confirmation of a positive result in a COVID-19 coronavirus diagnostic test triggered intense anxiety and stress about their long-term health on the one hand and the consequences of the disease on their children on the other.

“My first fear was what would happen to the children? Because after watching the others I was afraid that it might get serious. Even though I didn't have very severe symptoms, I was afraid of what would happen to my children, how the whole situation would turn out” (P1).

A significant number of respondents also noted that the main feeling of anxiety was compounded by fear. This fear was significantly intensified in people with underlying diseases, who were classified as high-risk groups.

“I was very scared because I happened to belong to a vulnerable health group” (P10).

*“… I'm an individual* with a lot of health problems... *I didn't know how this was going to turn out for me, ... I feared how it was going to turn out” (P12).*

The lack of trust in the traditional media combined with the absence of reliable and consistent information particularly regarding the coronavirus crisis, the outcome of the disease, personal protection measures, and vaccination caused confusion and increased participants' sense of uncertainty, fear, and sense of risk, thereby highlighting the need for effective health communication.

“There is a lot of misinformation and they have made us feel very nervous. Too, too much. They'd better stop. God forbid” (P1).

“At first the media scared me very much. I thought of the disease as a Hydra that was coming to devour me. And ... I was lying in bed terrified” (P4).

“It would be better and preferable for people not to watch the media. They only put out a fear with false news. There's the coronavirus, it's real, but I think this whole situation scared people too much. With a lot of lies ... First, they said you won't get sick, then they said you won't die. People who had coronavirus died and people who didn't died too. Vaccinated and unvaccinated, ... the media, the journalists are all directed” (P9).

In addition, the limited and planned telephone communication between patients’ relatives and nursing staff, as well as insufficient information, increased the sense of anxiety, psychological pressure, and stress in patients. The negative impact on their psychological state and health outcomes was further compounded by the fact that relatives had access to information about patients’ health conditions only through scheduled telephone communications and at specific times.

“I was asking how I was doing ... about the results of my tests ... the answers were typical ... which is what was happening in any medical briefing ... it was very little and in fractions of just a minute - no more” (P10).

“There was a specific timetable when family members could call for health updates, but nursing staff didn't pick up” (P8).

“I had no sense of the progress of my health. While I was feeling I was doing somewhat better the doctors were telling me that we'll see, you'll get out, and you'll get out, and you'll get out... However, my children were talking to the doctors at the briefing, and they were telling them that your mother's psychology is not good so that's why she's not improving …” (P6).

“When I was asking questions to both the doctors and the nursing staff, they avoided talking to me and providing me with any details... All they would tell me was we'll see, we'll see, we'll see, we'll see. Which made me even more anxious, since I had no idea how my health was progressing” (P12).

At the same time, the diagnosis of COVID-19 caused high levels of psychological distress in participants associated with a lack of reliable information about the progression of the disease and its future health implications. This reaction was attributed to the lack of personal contact and the feelings of tension, fear, anxiety, and stress induced after the diagnosis.

“I panicked at first because of my health condition. If it hadn't been like that, I think I wouldn't have been so scared, I would have been more comfortable. We'd been influenced that vulnerable groups were at risk” (P10).

“I was frightened by this uncertainty. That no one knew what it was. Everyone was confused... the doctors, the nurses, the state... All this confused me and reinforced my fears about what was to come” (P3).

“It was a disease that was affecting all the people around. At first, we didn't know how you could get infected. It wasn't something that was known. Even the doctors at the time were investigating it” (P2).

Category 3: causal attributions and meaning-making

This category includes the following two subcategories: (a) internal attributions and (b) external attributions.

An interesting finding of the study was that, despite the implementation of personal protective measures and public health precautions, the mode of infection was suggested to be the existence of factors that were not effectively controlled. Considering this, participants expressed the view that they were infected with the virus either through carelessness or exposure to an infectious person in the wider social environment (beyond the family).

“...I didn't really watch out; I didn't take it seriously...” (P1).

“I got infected because of my uncontrolled contact with people in my general environment” (P10).

A significant proportion of participants attribute their infection to their workplace, despite strict adherence to general hygiene precautions to stop the transmission of infection (e.g., hand washing, use of masks and antiseptics, ventilation, and vaccination).

“I believe I was infected from my workplace, in which a colleague in the office did not know he had the virus. I reckon I got infected through my contact with him” (P11).

“I was infected because of my professional activities” (P7).

“I got infected by a nurse” (P4).

In addition, a significant number of participants attributed their infection to third parties, expressing the belief that although they were following the social imperatives by applying the preventive measures against the spread of COVID-19 in their entirety (except for vaccination), they were infected by people who did not observe the necessary precautions and protection measures.

“I was very careful, ... I got infected in my workplace. Other people weren't watching out. Even though I was careful, I was also looking after others. I got infected because somebody was not careful - he was not taking the safety and protection measures. So, I realized that it was not only about the measures that I was taking. That's why I was infected” (P12).

Of particular interest is the perspective that the infection was a powerful test of faith in God: *“I believe it was God-given” (P2).*

The opinion on the origin of the coronavirus is also of interest. Some participants argued that the virus did not emerge through the process of natural selection (meaning, it did not come from any known coronavirus), which suggests that, in their opinion, it is artificial as it was deliberately created or modeled in a laboratory to serve personal self-interest or in the context of experimental testing.

“I believe that this was purely political. This was made by some people. I don't know who they are. They wanted to test the world. They wanted to see something. But it wasn't normal” (P9).

Category 4: toward mental resilience

Participants reported three main sources of support that helped them deal with the situation: (a) spiritual/religious beliefs; (b) personal resources of mental resilience, positive psychology, and the individual's ability to adapt in the face of adversity, to cope effectively with it, and to recover from it; and (c) help from health professionals.

Faith in God and religious practices, such as turning to religious figures (Saints and deified Fathers of the Christian Orthodox Church) and prayer, including viewing the illness as a spiritual test imposed by God to test one's faith or fortitude, were important coping mechanisms for many participants.

“I had faith. Some Fathers of the Christian Orthodox Church were calling me. I also had faith in God. I believed that if God decides something, I can't do anything else. It was God related. Whatever God decided” (P2).

“Faith in God helped me with love for my fellow man. Therein lies the secret. When I entered the ward, my friend didn't expect that I would be hospitalized too, nor did he know if I was sick. Since he was having a very hard time at the time, he was a little more sensitive. When he saw me entering the ward with the wheelchair, I saw his eyes as if he was seeing his savior” (P9).

“I was supported by my faith in God and the picture of the Virgin Mary that was in the ward” (P10).

Participants demonstrated significant mental resilience, manifesting internal strategies for dealing with the disease and vulnerability. Typical examples include handling and suppressing negative thoughts and emotions, self-restraint, sobriety, cultivating a positive attitude toward the future, and maintaining optimism for a speedy recovery.

“I drew strength from the effort to have a good psychology - not to feel discouraged. I decided that while being a young man, I would manage it and that I would not have any more problems. I would face them with every possible effort” (P11).

“I drew strength from me” (P5).

Several participants highlighted the beneficial effect of social support from the wider environment on their health recovery efforts. Messages of support, heartfelt expressions of compassion, and communication, whenever possible, with close family and friends, as well as interactions between health professionals and beneficiaries, led to positive outcomes, such as modification of their subjective interpretation and evaluation of their perceptions of their disease.

“From my children and from my sister, who supported me very much” (P4).

“I was supported by the friendly support of fellow doctors, nurses, and nursing staff. I was impressed by the generosity and supportive attitude of people I did not expect” (P7).

*“Because the nurses and doctors had all the mood of real offering with a big smile, a real hug for the sick person. It radiated even despite the harsh clothing they wore. Because it really radiated internally and they had this inner disposition to help the sick person in his/her true dimension. No fakes. I saw the inner joy of helping people. May they have strength and truly be able to always radiate that inner smile, which gives a great radiance to the sick, and hope to be able to balance their day and hold on to it to move forward*” *(P2).*

The emotions experienced by the participants during their illness and hospitalization had a significant impact on dealing with the illness. Research findings reveal a positive association between individuals' ability to handle their emotions, and, by extension, their need for psychological support, and personal factors such as their personality, level of psychological resilience, level of awareness, or perceptions of the disease.

It is worth mentioning that the low frequency of media exposure did not contribute to the creation of a climate of intense anxiety and fear about the disease; therefore, the participants faced the possible outcome of the disease with relative calmness and without panic attacks.

“I was unaware and not afraid. I was ignorant of the situation” (P2).

In contrast, the high frequency and systematic content of some participants' exposure to the media increased fear, and participants showed high levels of anxiety about their health outcomes.

“They made me nervous” (P10).

The primary goal and dominant thought/desire of most participants throughout their hospitalization was to wait for recovery and return to daily life.

“To get out of the hospital as soon as possible” (P1), (P2), (P3).

Spirituality, expressed through faith in God, enhanced the participants' emotional resilience and sense of inner peace throughout the disease. Despite any negative emotions that accompanied their hospitalization, religious faith provided them with relief and emotional support, helping them to manage them more effectively.

“I am strong, someone is protecting me, and I will make it” (P4).

“You do not fear anything with trust in God... when the grace of God visits you, you do not fear anything” (P9).

Finally, pushing away any negative thoughts, replacing them with positive ones, and having a positive outlook on coping with the challenges of the disease were directly linked to mental resilience and were reported as important factors of self-care and self-protection.

“I was trying to think positive and have a good psychology” (P11).

## Discussion

This study presents the psychological and social impact of COVID-19 and its association with negative memories experienced by patients 12 months after their hospitalization in COVID-19 treatment units of public hospitals. The findings demonstrated the multilevel impact of the disease. In addition to the known physical complications, the disease went beyond the narrow limits of acute disease and had a direct psychological impact on the participants, manifested mainly as anxiety, concern about the future, and uncertainty. It also led to indirect effects on the social life of the patients, such as isolation and social distancing. As a result, patients reported negative experiences, as their hospitalization was accompanied by the onset of a variety of symptoms that affected both their mental and social well-being.

More specifically, the mandatory isolation imposed on COVID-19 patients during their hospitalization, although effective in halting the pandemic [21], exacerbated the intensity of negative emotions (e.g., malaise, loneliness, boredom, insecurity, and fear of death) experienced by patients and increased their psychological distress, which confirms findings of previous studies [22,23] on the psycho-emotional effects of restraints during the pandemic. Indeed, the highly stressful environment created, with a predominant characteristic of a sense of isolation, combined with the feeling of uncertainty and loss of control experienced by COVID-19 patients, led to a significant deterioration in their mental health, causing a range of neurological or psychiatric disorders to emerge in a significant proportion of the population. The strong association between these and pandemic infectious diseases has also been demonstrated by findings of recent studies [24].

Similarly, in this study, participants emphasized that the experience of hospitalization would be unforgettable, given the profound psychological impact it had left on them. The social distancing and isolation they experienced resulted in the generation of a wide range of negative emotions, including feelings of vague pervasive anxiety, inability to relax, intense anxiety, anger, nervousness, fear, terror, and loneliness. Such feelings are common in cases of exclusion and social isolation and are also confirmed by a variety of studies.

At this point, it is worth pointing out that this feeling of detachment and the intense sense of fear, even after leaving the given social condition, as already reported by Brooks et al. [2], can lead to post-traumatic stress. This is partly due to the individual's fear of a possible repetition of similar events in the future with potentially even more severe consequences for the individual. In addition, the exposure of sufferers to uncertainty and excessive stress undermines the ability of COVID-19 patients to effectively cope with the negative emotions generated, causing feelings of insecurity, disorganization, and destabilization.

This insecurity, exposure to prolonged stress, and mental distress experienced by participants during the pandemic are also reflected in the research of Seehan et al. [25]. However, of particular interest is the finding that participants who reported strong religious faith were able to mitigate their negative emotions. Religiousness/spirituality, therefore, is recognized both as a stable predictor of the meaning of life and as a protective factor of the individual’s well-being, mitigating any negative effects of social isolation and uncertainty. These results are consistent with previous research confirming the positive effect of religiosity/spirituality on patients' mental health [26]. In the majority of cases, they provide evidence for positive effects of religiosity in patients with infectious and non-infectious diseases, rather than negative impacts.

At this point, it is worth noting that the results of Søvold et al.'s [27] study of COVID-19 patients after hospitalization revealed that religiosity/spirituality was the primary context in which sufferers interpreted the meaning of the disease. Despite the difficulties experienced, a significant proportion of participants reported improvement/development of their mental resilience. Similarly, the participants of this study reported that they felt graced and blessed that God enabled them not only to effectively face this new health challenge and cope with the disease but also to grow, develop, and discover new sources of strength while remaining true to their values. Their faith in God and social support were the root cause. These factors, including positive psychology, were important personal resources and helped them to face the disease with optimism while experiencing a sense of personal growth [26].

These people used their disease to redefine their lives and reframe the meaning of their social roles. In the same context, the research also highlights how the participants' optimism is closely linked to their personality, their positive attitude toward life, and the support they received from their environment. This characteristic, in the majority of existing studies, is found in participants with a strong personality, those with a positive approach to life, and those who are supported by their loved ones [27]. These findings are fully in line with previous research, such as that of Chen and Bonanno [3], which highlights the importance of optimism and having a strong social network in promoting positive mental health and well-being [28]. One possible explanation for the strong correlation between participants' personal resources and mental well-being may be that these individuals are trying to change their perspective on life by creating a more meaningful future and deriving their life purpose from the existence of challenges.

However, along with the patients who treated the ordeal of illness as a springboard to find meaning in their lives and develop spiritually, there was also a significant number of those who were mentally burdened by the disease. In this case, the researchers consider the health complications of the coronavirus, restrictive measures, and generalized fear due to misinformation in the media to be the causes of this phenomenon.

Indeed, findings from recent studies reveal that patients with COVID-19 and underlying diseases showed a fairly high level of increased susceptibility to mental disorders. In the same context, studies by Thomson et al. [29] and Seehan et al. [25], respectively, demonstrate that subjective perception of disease severity constitutes a fundamental pillar in the deterioration of the individual's mental state. However, according to the reports of the participants of the present study, the high levels of mental exhaustion, depressive symptomatology, uncertainty about the future, feelings of death and giving up on life, lack of hope, manifestations of post-traumatic stress, sadness, confusion, and anger experienced during their stay in the COVID-19 treatment units intensified significantly.

Our findings, in full agreement with those of Torales et al. [30], confirm the results of related studies conducted during the pandemic [31] and highlight the systematic occurrence of a wide range of anxiety, depressive, confusional, and psychiatric symptoms in patients with COVID-19 hospitalized in special treatment units. In more detail, after their hospitalization, participants reported feelings of lack of meaning, mental distress, feelings of emptiness, uncertainty, irritability, and lack of confidence, which significantly affected their health-related quality of life (HRQL) in the short and long term [15].

The existence of long-term psychological and social consequences, such as fear of health or fear of a recurrence of similar situations, the adoption of excessive protective measures even when there is no real risk, anxiety, uncertainty about the future, grief, a significant reduction in social contacts, and detachment can lead to a significant deterioration in a person's quality of life. Therefore, the need for the development and implementation of specialized psychological interventions is deemed necessary for the recovery of their mental health. Through specialized psychological support, patients will explore their feelings, process their experience of hospitalization, and develop individualized coping mechanisms that respond to each individual's unique needs and experiences empowering them to cope effectively by improving their HRQL.

In conclusion, it is worth noting that the roots of the pandemic's long-term psychological and social effects can also be found in the social inequalities that were intensified by the pandemic, leading to stigmatization [32], especially of specific population groups. A typical example is the stigmatization experienced by unvaccinated patients, both during hospitalization, which manifested through differential treatment by the social environment, healthcare providers, and the State itself, and also after their discharge from the healthcare institution. The discrimination suffered by the unvaccinated has caused long-term negative effects on their mental and social health, significantly affecting their HRQL. This difference in treatment may, of course, be due to several factors, such as the personal beliefs of staff or hospital policy. In any case, according to the study by Woong et al. [33], the social stigmatization associated with COVID-19 has serious negative effects on an individual’s mental health and may worsen the outcome of the disease. To effectively address this problem and to provide comprehensive and personalized care to patients, close and effective collaboration and communication between medical, nursing, social, and mental health staff is recognized as vital [1].

Limitations

The qualitative study design with a small sample size is a limitation of the present study that does not allow for the generalization of the findings. In addition, the sample consisted of participants from one specific region of Greece, which can also be considered a limitation. Finally, the psychological state of the participants at the time of the interviews may have influenced their responses, leading to either exaggeration or understatement of their experiences.

## Conclusions

The study revealed a significant impact of the COVID-19 pandemic on the mental and social health of patients after being infected with the coronavirus and hospitalized in specialized treatment units. It was observed that factors such as uncertainty about disease progression, a sense of losing control, and social stigma contributed to the deterioration of psychological well-being, especially in vulnerable groups, such as individuals with underlying health conditions or those who were unvaccinated. An interesting finding relates to the role of religion as a protective factor; patients with deep religious faith reported higher levels of resilience and psychological well-being. However, this study faced methodological challenges in assessing the psychological state of the participants, particularly regarding the reporting of negative emotions.

Overall, the findings highlight the need for a multidisciplinary approach to understand and address the long-term psychosocial impact of the pandemic. Further quantitative and qualitative research is suggested to identify the biopsychosocial mechanisms linking COVID-19 to psychological disorders and to develop tailored prevention and treatment interventions.
